# Changes in alkaline band formation and calcification of corticated charophyte *Chara globularis*

**DOI:** 10.1186/2193-1801-2-85

**Published:** 2013-03-05

**Authors:** Chika Kawahata, Masumi Yamamuro, Yoshihiro Shiraiwa

**Affiliations:** 1 Graduate School of Frontier Sciences, The University of Tokyo, Kashiwanoha, Kashiwa, 277-8573 Japan; 2 Graduate School of Life and Environmental Sciences, University of Tsukuba, Tsukuba, 305-8572 Japan

**Keywords:** *Chara corallina*, *Chara globularis*, Alkaline band, Acid band, SEM

## Abstract

Calcification by charophytes improves the quality of water, although most studies on calcification have only examined ecorticate species. We investigated the formation and relationship of alkalines and acids with regard to calcification on internodal cells in *Chara corallina*, an ecorticate species, and *Chara globularis*, a corticate species. We observed that alkaline and acidic areas with distinct banding patterns form on the internodal cells of *C*. *corallina*. The entire periphery of internodal cells was alkalized, and no distinct acidic area developed in *C*. *globularis*. By electron microscopy of these internodal cells, the calcified areas occurred primarily in alkaline areas with a banding pattern in *C. coralline*. However, phenomenon also occurred homogeneously inside of the entire cortex and cell wall in *C. globularis*. We also investigated the formation and relatiohship of alkalines and acids with regard to calcification on internodal cells of various ages from a single thallus of *C. globularis.* For internodal cells of *C. globularis*, a uniform calcified area lay between the cell wall and cortex on all cells, irrespective of age. In contrast, young cells bore an alkaline area that was uniform and widespread throughout their entire periphery, but the alkaline area in older cells was split into smaller segments in a banding pattern. Acidic areas were absent in young cells. These results indicate that the mechanisms by which alkaline and acid areas form differ in the presence and absence of cortex and between species.

## Introduction

Charophytes are aquatic, primarily freshwater green plants that are phylogenetically related to early land plants. Certain charophytes produce carbonate biominerals, which aggregate into significant deposits in lakes (Dean, 1981). Charophyte calcite encrustations are sometimes a major component of lacustrine carbonate deposits worldwide, frequently providing extended and continuous sequences of sedimentation throughout the Holocene period and beyond (Garcia, 1994).

The high, rapid biomass production and calcite incrustation of charophytes allow large amounts of nutrients to be absorbed from the water (Kufel and Kufel 2002). Increased sedimentation, a lower bioavailable fraction of phosphorus, and carbon limitation inside charophyte beds contribute to the clear water state (Blindow et al. 2002).

Charophytes are slightly (eg, *Nitella* species) or heavily (most *Chara* species) encrusted, and the morphology of the thallus (corticate or ecorticate) is a significant factor differentiation with regard to its biomineralization. The mechanisms of alkaline band formation and calcification in *Chara* have been studied primarily in cultur experiments using *Chara corallina* also known as *Chara australis* (Lucas, 1979; McConnaughey, 1991; Proseus et al., 2000. The correlation between alkaline and calcified areas has been demonstrated in *Chara braunii* (Okazaki and Tokita, 1988). These 2 species are known as ecorticates and share calcified area features.

Alkaline and acidic areas also have distinct banding patterns. However, little attention has been paid to the mineralogy and chemistry of charophyte biomineralization in corticate species, which might be due to specific differences in the mechanisms of mineralization in *Chara.* Thus, we focused on the physiological mineralogy of charophyte biomineralization in corticate species.

In this study, the formation of alkaline areas and calcification of internodal cells were compared. Differences in the presence and absence of cortex between species was investigated in 2 *Chara* species—*C. globularis*, a corticate species that lacks calcified areas in a banding pattern, and *C. corallina*, the species that has been studied most extensively.

## Materials and methods

### Plant materials

*Chara corallina* Klein ex. Willd. em. R.D.W. (identical to *Chara australis* R. Br.), an ecorticate species, was obtained from the cloned culture strain (NIES-1585), provided by the Microbial Culture Collection, National Institute for Environmental Studies. *Chara globularis*, a corticate species, was collected at Yuno Lake (N36°47’, E139° 25’), Tochigi prefecture, Japan, in September 2008. Both species were cultured for more than 2 weeks in 2-L culture bottles that contained 10 g of culture soil and 1.5 L of reverse-osmosis water at 15°C. The culture was illuminated by a fluorescent lamp at an intensity of 10 μmol m^-2^ s^-1^ with a light:dark cycle of 12 h:12 h.

To compare calcified areas, internodal cells of *C. globularis* that were recovered from bottom mud that was collected in October 2009 at Oito Pond (N33°44’, E130° 51’), Fukuoka prefecture, Japan, were also used in the observation experiment.

### Electron microscopy of calcium carbonate precipitates on internodal cells

Internodal cells of *C. globularis* and *C. corallina* were fixed overnight in 2.5% glutaraldehyde (pH 7.0) and freeze-dried (FDU-810, EYELA). Two types of samples were prepared. One was freeze-dried, embedded in epoxy resin (Petropoxy 154, Maruto), and ground on an ML-180 (Maruto) to flatten the sample’s surface. The other type was freeze-dried, and the internodal cells were attached directly to carbon tape. Both samples were subjected to carbon evaporation (Quick Carbon Coater SC-701C, Sanyu Denshi) and used for observation and analysis on a cold cathode field emission scanning electron microscope (SEM) (S-4500, Hitachi). Energy dispersive X-ray spectroscopy (EDS) and SEM were used to detect and map calcium.

### Alkaline band detection on internodal cells

Alkaline bands were detected per Okazaki and Tokita (1988) and Mimura and Shimmen (1994). Mature *C. corallina* and *C. globularis* internodal cells were isolated from different thalli, incubated for several days (light:dark = 12 h:12 h; 760 lux condition) in preculture solution (1 mM NaCl, 0.1 mM KCl, 0.1 mM CaCl_2_; pH 5.6), and left overnight in darkness. Then, the cells were embedded in 0.5% agarose gel (low gelling-temperature agarose, Wako) containing 0.2 mM NaHCO_3_, 10 mM CaCl_2_, and 0.1 mM phenol red and cultured for 30 min. The areas in which phenol red turned red and yellow indicated alkalized and acidified sections, respectively.

During culture, the cells were observed on a stereoscopic microscope (SMZ645, Nikon) 0, 2, and 6 hours after the start of the light period. For *C. globularis*, internodal cells of various ages were isolated from the same thallus, and similar procedures as above were conducted to check the age-dependent differences in alkaline bands on *C. globularis* internodal cells.

## Results

### Calcification on internodal cells of c. globularis

**Figure 1:**
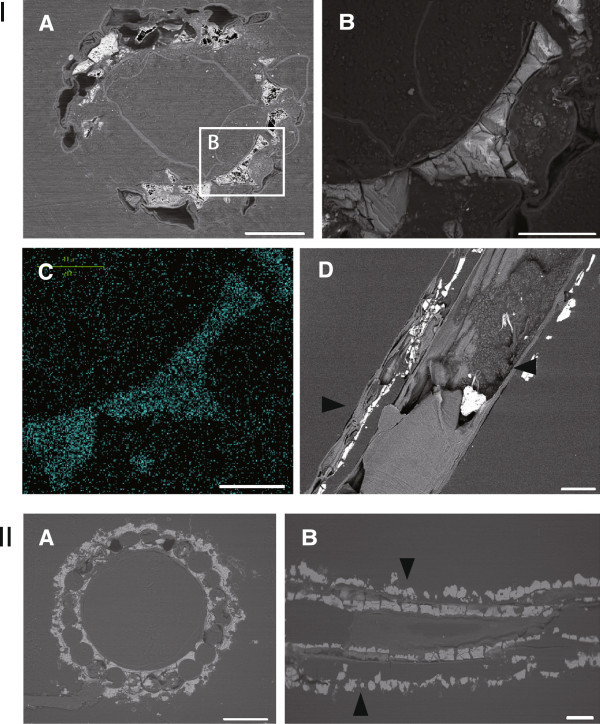
**Microscopic observation of calcification of an internodal cell of*C. globularis*from 2 lakes. I.** SEM micrograph (back scattered electron image) (**A, B** and **D**) and an EDS image for mapping calcium (**C**) a section of an internodal cell (**A**‐**C** and **D**: 18th and 21th from the tip of a thallus, respectively). Scale bars: 100 μm. **II.** SEM micrograph (backscattered electron image) of cross (**A**)- and longitudinal (**B**) sections of internodal cells (**A** and **B**: 8th and 6th cell from the tip of the plant, respectively). Scale bars: 100 μm. Samples from Yuno Lake (**I**) and Oito Pond (**II**). Arrows in **I**-**D** and **II**-**B** show an area of calcification.

In our instant elemental analysis of the white material by EDS and electron microscopy, we detected a high proportion of calcium (approximately 50%). Element mapping of calcium indicated that all white precipitates consisted of a large amount of calcium (Figure 1-I). Calcified areas lay outside and inside of the cortex, defined as an area in which the backscattered electron image was white and the calcium element was abundant (Figure 1-II).

### Difference in alkaline band formation between corticated and ecorticate species

**Figure 2:**
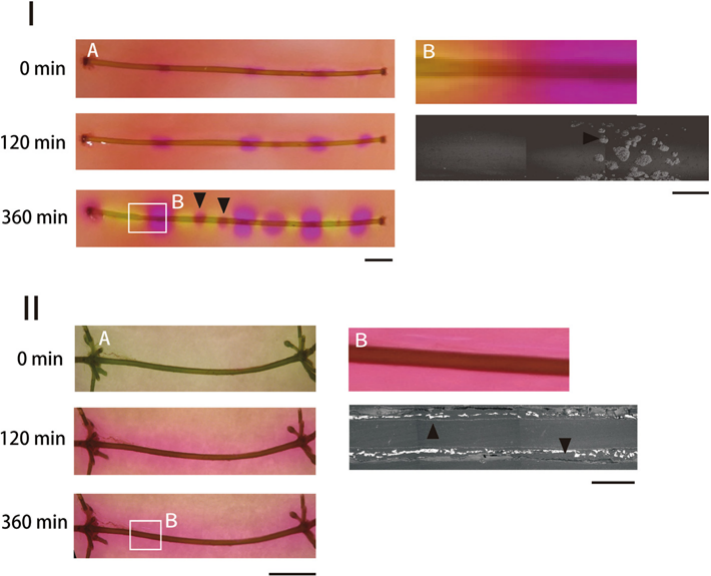
**Comparison of the formation of an alkaline area and calcification area in 2 charophytes. I**: 4th internodal cell of *C. coralline* (NIES‐1585), **II**: 6th internodal cell of *C. globularis*. **A** and **B**: Stereoscopic and SEM micrograph (back scattered electron image) of internodal cells, respectively. Scale bars, 5 and 0.5 mm in **A** and **B**, respectively.

The internodal cells of *C. corallina* formed alkaline and acidic areas in a banding pattern around the cells over time (Figure 2-IA); some alkaline areas developed after 6 h of irradiation (Figure 2-IA, arrows). In contrast, the entire periphery of internodal cells in *C. globularis* turned red. Alkaline and acidic areas with banding patterns were not detected in this species (Figure 2-IIA).

Calcium carbonate precipitates formed on internodal cells on which an alkaline area was observed in *C. corallina* (Figure 2-I, arrows). Calcified areas were observed outside of the cell wall of *C. corallina* internodal cells but were uniform between the cortex and cell wall of *C. globularis* internodal cells of (Figure 2-IIB).

### Age-dependent differences in alkaline bands on internodal cells of *C*. *globularis*

**Figure 3:**
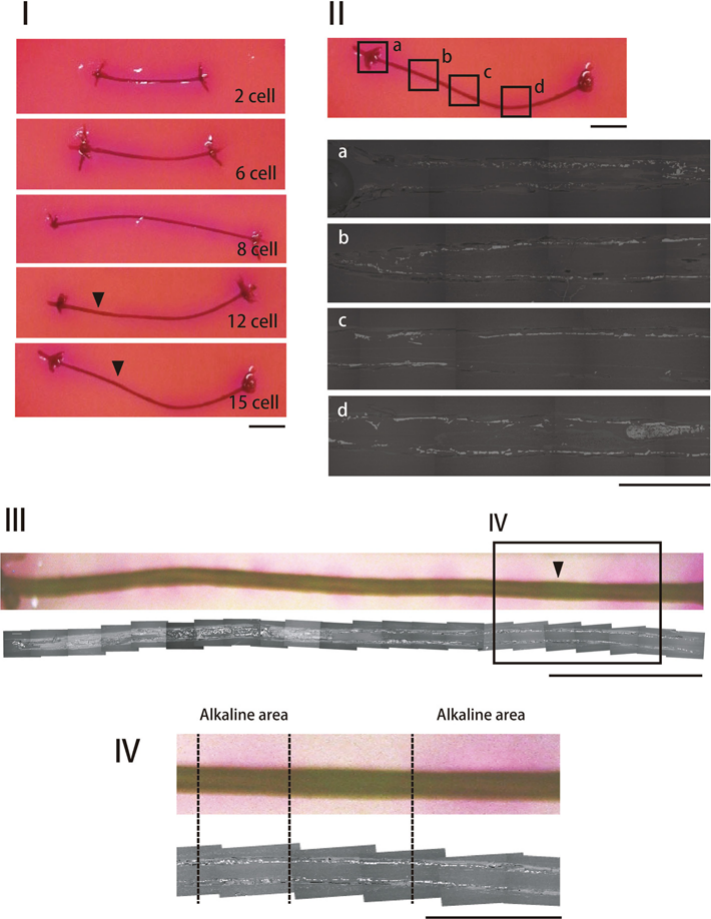
**Change in formation of alkaline and calcification areas on internodal cells with different ages in*C. globularis*. (collected from Yuno Lake) I.** Stereoscopic micrograph of internodal cell (2, 6, 8, 15th cell from the tip of thallus). **II.** Stereoscopic and SEM micrographs (back-scattered electron image) of 15th internodal cell (**a**‐**d**). **III** and **IV**. Stereoscopic (above) and SEM micrograph (below) of the 23th internodal cell at low and high magnification, respectively. Scale bars: **I**‐**III**, 5 mm; **IIa**‐**d** and **IV**, 1 mm.

Alkaline and calcified areas were observed on internodal cells with various ages in a single thallus of *C. globularis*. Alkalization was higher in younger cells than older cells. An alkalized area was observed in the entire periphery of young internodal cells, which lay in the upper section of the thallus (internodal cells at the 2nd, 6th, and 8th positions from the top) (Figure 3-I).

In contrast, band formation of the alkalized area was not patent in old internodal cells, which were located in the lower part of the thallus (internodal cells at the 12th and 15th positions from the top) (Figure 3-I). By electron microscopy, calcium carbonate precipitates were widespread between the cortex and cell wall. Precipitates were also detected in areas in which alkalization was absent from around the cells (Figure 3-II).

Alkaline band detection and electron microsopy were also performed on an internodal cell at the 23rd position from the top from another individual. Alkaline areas were observed in a banding pattern. Although the border was indistinct, acid areas were not clearly noted (Figure 3-III, IV). As on the internodal cell at the 15th position from the top (Figure 3-II), calcium carbonate precipitation was observed uniformly in all gaps between the cortex and cell wall, irrespective of where the alkaline area lay (Figure 3-IV).

## Discussion

Environmental Ca^2+^ reacts with bicarbonate or carbonate ions to produce calcium carbonate outside of the cell wall of charophytes. Such calcification is believed to develop with a banding pattern due to the alkaline band in which bicarbonate/carbonate ions dominate (Okazaki and Tokita, 1988).

Calcification and alkalization mechanisms in charophytes have been studied using various methods with regard to photosynthesis and alkaline band formation—namely, by changing the extracellular pH (Lucas, 1979) and determining the CO_2_ species that are absorbed by an internodal cell (Shiraiwa and Kikuyama, 1989; Wayan et al., 1994). For example, 3 to 5 calcified areas within a banding pattern, detectable to the naked eye, are present in a single internodal cell of *C. braunii*, an ecorticate species.

However, in our study, punctuated spots of calcium carbonate without a banding pattern were observed outside of the thallus of *C. globularis,* a corticate species, from Yuno Lake. By electron microscopy, we also confirmed that the entire exterior of the cortex was calcified. With respect to the calcification of the cortical exterior of the thallus in *C. globularis*, calcified areas were uniform over the internodal cell inside and outside of the cortex in the cross-section. Despite some deviations between samples from various sites (Figure 1), such a pattern is unique, because calcification usually occurs in a banding pattern in charophytes.

The distinct alkaline and acidic banding patterns on *C. corallina* were internodal, as in *C. corallina* (Mimura and Shimmen, 1994). In contrast, in *C. globularis*, a corticate species, the entire cell periphery was alkalized in young internodal cells: the alkaline area began with the growth of alkaline and acid bands in old cells. These findings support the hypothesis that calcification occurs in the alkaline area. Calcification on old internodal cells of *C. globularis,* however, occurred but not in where alkaline areas developed.

The mechanism by which alkaline areas form can be distinguished in the presence and absence of cortex, although the function of the cortex in alkaline band formation is unknown. We have demonstrated that alkalization and calcification are induced over the entire cell in *C. globularis*. The alkaline areas that are formed on the surface of internodal cells in charophytes are generally developed by local OH^-^ efflux or H^+^ influx (Lucas, 1979).

It is believed that local OH^-^ efflux (or H^+^ influx) of *C. corallina* internodal cells is regulated by protoplasmic streaming on the development of partial alkaline areas, and the system for OH^-^ efflux (or H^+^ influx) is distributed over the entire cell membrane (Lucas and Dainty, 1977; Lucas and Shimmen, 1981). The electrophysiological properties of internodal cells in *C. globularis* differed from those of *C. corallina* (Shimmen, 1994); thus, such differences in regulatory mechanism of OH^-^ efflux (or H^+^ influx) are due to disparities in alkaline band formation in *C. globularis* versus *C. corallina*.

How inorganic carbon species, such as CO_2_ and HCO_3_^-^, are incorporated into charophyte cells is unknown, despite many studies examining such mechanisms (Lucas, 1976; Lucas, et al., 1978; Walker, et al., 1980; Shiraiwa and Kikuyama, 1989; Mimura et al. 1993). The involvement of carbonic anhydrase (CA) has been reported in *Chara* (Price et al., 1985; Ray et al., 2003). Algal CA is believed to be required for effective use of inorganic carbon as an alkaline when *Chara* cells use CO_2_ as an inorganic carbon source (Shiraiwa and Kikuyama, 1989).

Alkalization of the medium triggers a decrease in free CO_2_ concentration due to the shift in CO_2_/HCO_3_^-^ equilibrium. Because lower CO_2_ concentrations induce CA, which increases CO_2_ uses by cells, the affinity of charophyte cells to CO_2_ is elevated. As a result, the incorporation of CO_2_ is enhanced in alkalized areas.

In our examination of the development of alkaline and calcified internodal cell areas of *C. globularis,* certain parts of the periphery of old internodal cells were free of OH^-^ ions. However, uniform carbonate assimilation was observed between the cortex and cell wall in all internodal cells. Smaller alkaline areas were also noted outside of the oldest internodal cell (23rd cell), in contrast to the alkaline areas in the 15th cell from the tip of the thallus (Figure 3-I, II). However, unclear acidic areas were observed where alkaline cell areas were absent from younger and older internodal cells.

Because alkaline areas cover the entire periphery of young internodal cells in *C. globularis*, calcification is induced and develops there, including the cell wall and the cortex. Whereas old cells bore unclear acidic bands and separate alkaline areas, continuous calcified areas were observed over the entire cell (Figure 3-IV). Such unclear acidification might be due to the neutralization of acid by CaCO_3_ precipitates, whereas weak alkalization areas can be observed easily. Thus, the entire area of internodal cells was alkalized in *C. globularis* when the internodal cells were calcified. Calcification was also observed under the cortex of all internodal cells, likely because the lime that was formed during these younger cell stages remained between the cell wall and cortex. Further calcification occurred in the form of a preexisting crystal.

The natural crystallization of calcium carbonates is believed to be affected by various factors, such as inorganic matter, temperature, and pH of water, as well as organic matter, and how agitated the water is in the system (Kitano, 1962). Further, calcification in charophytes is affected by plant age, photosynthetic activity, calcium and carbonate ion concentration, and pH and temperature in the habitat (Smith, 1985). Our study demonstrated that more carbonate precipitated in old *C. globularis* cells from Yuno Lake and Oito Pond; calcification sites had spread inside and outside of the cortex (Figure 1-II, Figure 2-II). These findings indicate that the formation of an alkaline area was inhibited outside of the cortex, because the inside was covered in calcium carbonate precipitate. This pattern might be the reason why unclear acidic bands and separate alkaline areas were observed more frequently in the extracellular space of old *C. globularis* internodal cells (Figures 2, 3).

Our findings suggest that the mechanisms of calcium carbonate mineralization differ between *Chara* species. Further study is needed to gain a systematic understanding of charophyte mineralization, which can be the basis of phytoremediation of lakes using charophytes.
